# Factors associated with serious adverse event following immunization: a real-world analysis of the vaccine adverse event reporting system 2025

**DOI:** 10.3389/fpubh.2026.1820862

**Published:** 2026-04-29

**Authors:** Xuejiao Pan, Yaping Chen, Fuxing Chen, Hanqing He, Xiaohua Qi, Hui Liang

**Affiliations:** Department of Immunization Program, Zhejiang Provincial Center for Disease Control and Prevention, Hangzhou, China

**Keywords:** adverse events following immunization, clinical immunology, pharmacovigilance, vaccine adverse event reporting system, vaccine safety

## Abstract

**Background and aims:**

While vaccination is a vital and cost-effective public health tool, a small fraction of recipients experience serious adverse events (SAEs) that impose a profound clinical and systemic burden. Identifying the specific factors that predispose an individual to an SAE remains complex due to limitations in existing literature. This study analyzes SAE following immunization in the US population and identifies associated factors.

**Methods:**

We analyzed a national cross-section of 50,655 reports submitted to the Vaccine Adverse Event Reporting System (VAERS) throughout 2025. Data were categorized into five domains: demographic characteristics, clinical features, medical history, vaccine variables, and patient outcomes. A multivariable logistic regression model was employed to identify factors associated with reporting SAEs.

**Results:**

Of the 50,655 AEFIs reported (median age: 40 years), 9.58% were classified as SAEs. Multivariable analysis identified significant demographic and clinical factors associated with SAE reporting, including advanced age (≥65 years, aOR: 1.73), male sex (aOR for females: 0.72), longer onset times (aOR: 1.06), administration facilities (military, aOR: 1.76; private facilities, aOR: 1.39), and systemic symptoms (aOR: 1.72), while local symptoms were highly protective (aOR: 0.35). Patient history variables, such as current illness (aOR: 1.51) and medical history (aOR: 1.24), were associated with increased odds of SAE reporting, while prior reactions were protective (aOR: 0.77). Regarding vaccine characteristics, mRNA platforms (Pfizer/Wyeth aOR: 1.65), second doses (aOR: 1.33), non-intramuscular administration routes (oral aOR: 2.85; subcutaneous aOR: 1.51), and right-arm injections (aOR: 1.24) were all associated with higher odds of SAEs.

**Conclusion:**

SAE reporting is driven by a complex intersection of demographic, clinical, and vaccine-related characteristics. These findings carry significant clinical and public health implications, highlighting the need for enhanced screening and proactive monitoring of high-risk populations to optimize global vaccine safety protocols. Future studies should integrate passive reporting with active surveillance to optimize vaccine safety protocols.

## 1 Introduction

Vaccination is a critical, cost-effective public health intervention, preventing an estimated 3.5 to 5 million deaths annually (1). By mitigating the burden of over 30 infectious diseases, immunization programs have been pivotal in achieving a 40% reduction in global infant mortality over the past 50 years (1). However, as with all medical interventions, vaccines can be associated with adverse events following immunization (AEFI) (2). An AEFI is defined by the World Health Organization (WHO) as “any untoward medical occurrence that follows immunization and which does not necessarily have a causal relationship with the usage of the vaccine.” (3) Globally, the reported incidence rates of AEFIs exhibit significant heterogeneity, driven by a complex interplay of geographic factors, vaccine platform characteristics, and the structural differences between active and passive surveillance systems (2, 4). Notably, these events are predominantly mild and transient local or systemic reactions that typically manifest within the first seven days post-vaccination (2, 4).

While most AEFIs are mild, a small fraction of vaccine recipients may experience serious adverse events (SAEs), defined as those requiring hospitalization, causing permanent disability, or resulting in death (5). Globally, the incidence of SAEs remains remarkably low. For instance, a study reported that only 17 out of 1,053 children (2%) reported an SAE (6). However, their impact is profound, extending beyond individual clinical risks to encompass significant systemic burdens, including the necessity for intensive medical intervention and long-term rehabilitative care (7, 8). Furthermore, even rare SAEs can disproportionately increase vaccine hesitancy, leading to decreased immunization coverage and the potential resurgence of vaccine-preventable diseases (9). In the United States, declining MMR coverage has already been linked to a resurgence of measles, with confirmed cases in early 2025 nearly five times higher than the previous year (10). Furthermore, modeling suggests that if current declines in childhood vaccination rates continue, previously eliminated diseases like measles, rubella, and polio could return to endemic status in the U.S. within the next few decades (11). Consequently, monitoring SAEs is essential for sustaining the public trust necessary to maintain herd immunity (12).

Despite the importance of identifying SAEs, the factors that predispose an individual to a serious rather than a non-serious outcome remain complex (2, 12, 13). Previous research has suggested that demographic variables, specific vaccine types, and pre-existing medical conditions may influence the severity of adverse reactions (2, 12, 13). However, existing literature is often limited by methodological constraints. Many studies rely on pre-licensure trials with small sample sizes and short durations or focus on non-representative populations. Additionally, contemporary analyses are frequently narrow in scope, often prioritizing COVID-19 over broader immunization trends. Furthermore, most research also lacks a comprehensive overview that simultaneously integrates demographics, clinical presentations, and diverse vaccine platforms.

To address these existing research gaps, this study utilizes the Vaccine Adverse Event Reporting System (VAERS) to analyze a comprehensive national cohort of AEFI reports within the United States (14). It is important to note that VAERS is a passive surveillance program, which relies on individuals to voluntarily report experiences. Consequently, data within this system represent reporting patterns and potential safety signals rather than measures of true clinical incidence or definitive establishment of causality. Understanding these reporting associations, however, is vital for identifying trends that warrant further controlled investigation. Specifically, we focus on SAEs through a multivariable framework that simultaneously integrates demographic characteristics, clinical presentations, baseline patient health profiles, administrative settings, and vaccine-specific factors. By identifying factors associated with SAE reporting, this research aims to provide actionable clinical insights to enhance the safety monitoring and public health profile of both routine and emergency-authorized immunizations.

## 2 Methods

### 2.1 Data source

Data were extracted from VAERS, a national spontaneous (passive) surveillance system co-administered by the Centers for Disease Control and Prevention (CDC) and the Food and Drug Administration (FDA) (14). To ensure the inclusion of contemporary safety signals and maintain maximal data comprehensiveness, we analyzed a cross-section of all reports submitted to the VAERS database for the full calendar year 2025. VAERS serves as an early warning system to monitor vaccine safety post-licensure or post-authorization in the United States (15). It enables continuous monitoring of vaccines in a diverse, real-world population across millions of administered doses. The database consists of publicly available, de-identified, and anonymous reports submitted by vaccine manufacturers, healthcare professionals, and members of the public (including patients and caregivers) (16). Healthcare providers are mandated by law to report certain adverse events, while manufacturers are required to report all AEs brought to their attention.

The VAERS database is organized into three distinct datasets: one containing patient demographics and clinical outcomes, one for vaccine-specific information (e.g., manufacturer and lot number), and one for clinical symptoms. Clinical signs, symptoms, and outcomes are coded by professional staff using the Medical Dictionary for Regulatory Activities (MedDRA). These terms are organized hierarchically into System Organ Classes (SOCs) and Preferred Terms (PTs). A single report may be assigned multiple PTs, which represent clinical impressions rather than confirmed medical diagnoses. For this study, all symptoms were mapped to the most recent MedDRA version (v27.0), and duplicate entries—identified by unique VAERS IDs—were consolidated into a single record to ensure data integrity.

### 2.2 Measures

To analyze the reported adverse events, variables extracted from the VAERS database were categorized into five primary domains:

#### 2.2.1 Demographic characteristics

Data included sex (male, female, unknown), geographic region, and age. Geographic data were standardized by recoding the reported U.S. states and territories into five primary regions (Northeast, South, Midwest, West, and Territories), with an additional ‘Unknown’ category assigned to reports with missing or indeterminate location data. Age was evaluated both as a continuous variable and by category, classified into four groups: pediatric ( 〈18 years), adult ( 18–64 years), older adult ( ≥65 years), and unknown.

#### 2.2.2 Case and clinical characteristics

Case characteristics included onset time (days from vaccination to symptom onset), administration setting (e.g., Pharmacy, Private Clinic, Military Facility, Senior Living Center), and diagnostic or laboratory tests (Yes/No). Clinical symptoms included injection-site reactions (e.g., localized pain, redness, or swelling) and systemic reactions (e.g., fever, fatigue, or headache), as defined by MedDRA Preferred Terms.

#### 2.2.3 Patient medical history

Baseline health status was assessed using binary (Yes/No) indicators for concurrent medications, current illness, significant medical history, prior adverse vaccine reactions, and allergies.

#### 2.2.4 Vaccine characteristics

Indicators included vaccine type (classified as COVID-19 or routine non-COVID, such as influenza, HPV, and varicella), vaccine manufacturer (e.g., Pfizer/Wyeth, Moderna, Merck), dose number (e.g., dose 1, dose 2, or booster), vaccination route (e.g., intramuscular, oral, subcutaneous), and vaccination site (e.g., left arm, right arm, nasal).

#### 2.2.5 Case severity and patient outcomes

Specific patient outcomes tracked included healthcare utilization (clinic or emergency room visits, hospitalization) and severe clinical endpoints (permanent disability, life-threatening illness, or death). Reports were dichotomized into serious and non-serious cases based on the Code of Federal Regulations (21 CFR § 600.80) (5). A report was classified as a SAE if it met at least one of the following non-mutually exclusive criteria: death, life-threatening illness, permanent disability, inpatient hospitalization (or prolongation of existing hospitalization), or congenital anomalies (5). Because a single report may involve multiple serious outcomes, these categories were analyzed as overlapping rather than mutually exclusive variables.

### 2.3 Statistical analysis

Statistical analyses were conducted using a three-stage approach to identify factors associated with SAEs. First, descriptive statistics were used to summarize baseline characteristics, with categorical variables reported as frequencies and percentages, and continuous variables (e.g., age, onset time) presented as medians and interquartile ranges (IQRs) due to non-normal distributions. Second, bivariate comparisons between serious and non-serious reports were performed using Pearson’s Chi-square tests for categorical data, and independent t-tests or Mann–Whitney U tests for continuous data. Third, a multivariable logistic regression model was constructed to determine factors independently associated with SAEs. Initially, a significance threshold of *p* < 0.10 in the bivariate analysis was used to determine inclusion in the multivariable model. However, given the large sample size (*N* = 50,655), all examined variables reached statistical significance (*p* < 0.001 in nearly all cases). Therefore, a full-model approach was adopted, where all candidate variables were entered into the multivariable logistic regression. This approach ensured comprehensive adjustment for all conceptually relevant factors—such as age, sex, and vaccine type—thereby minimizing the risk of omitting important confounders and avoiding the instabilities associated with purely data-driven stepwise selection. To ensure the validity of our regression estimates, multicollinearity was assessed using Variance Inflation Factors (VIFs); all included variables had VIFs < 5, indicating no significant multicollinearity. Results were reported as adjusted odds ratios with 95% confidence intervals, and model fit was assessed via the Hosmer-Lemeshow test. Statistical significance was defined as a two-tailed p -value 〈0.05 . All analyses were performed using STATA software (version 17.0).

### 2.4 Declaration of Generative AI use

During the preparation of this manuscript, the authors used Gemini (version 1.5 Flash, Google) to assist with structuring the abstract and cover letter, refining technical prose, and organizing and summarizing statistical analysis findings for clinical presentation. This tool was employed to ensure adherence to journal formatting standards and to enhance the clarity of the research findings. Following the use of this AI tool, the authors independently reviewed and edited the content to ensure accuracy. The authors take full responsibility for the integrity of the data and the final interpretation presented in the manuscript.

### 2.5 Ethical considerations

This study utilized de-identified, publicly available data and was therefore deemed exempt from Institutional Review Board (IRB) review under 45 CFR 46.104. Informed consent was also not required because the analysis was retrospective and used de-identified data. The research was conducted in accordance with the tenets of the Declaration of Helsinki and applicable federal laws regarding the use of public surveillance data.

## 3 Results

### 3.1 Demographic and temporal distribution

**Figure 1:**
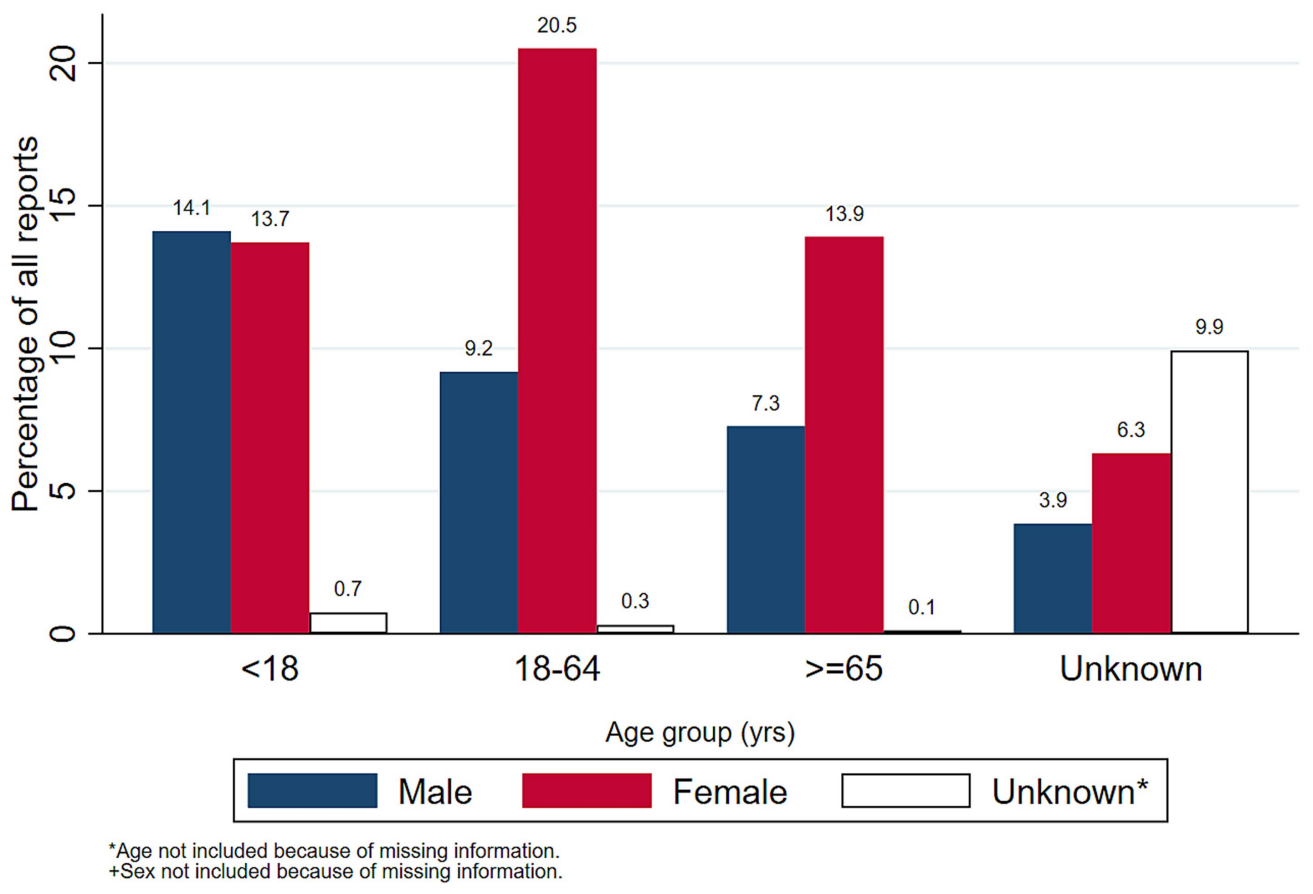
Distribution of VAERS reports by age group and sex (*N* = 50,655). VAERS, vaccine adverse event reporting system. Bars represent the percentage of total reports within each age category, stratified by sex: male (Navy), female (Cranberry), and unknown (Hollow/White). Age groups are defined in years. * Unknown represents age or sex not included because of missing information. Note that the “Unknown” age category includes reports in which patient age was not recorded, accounting for approximately 20.1% of the total sample.

**Figure 2:**
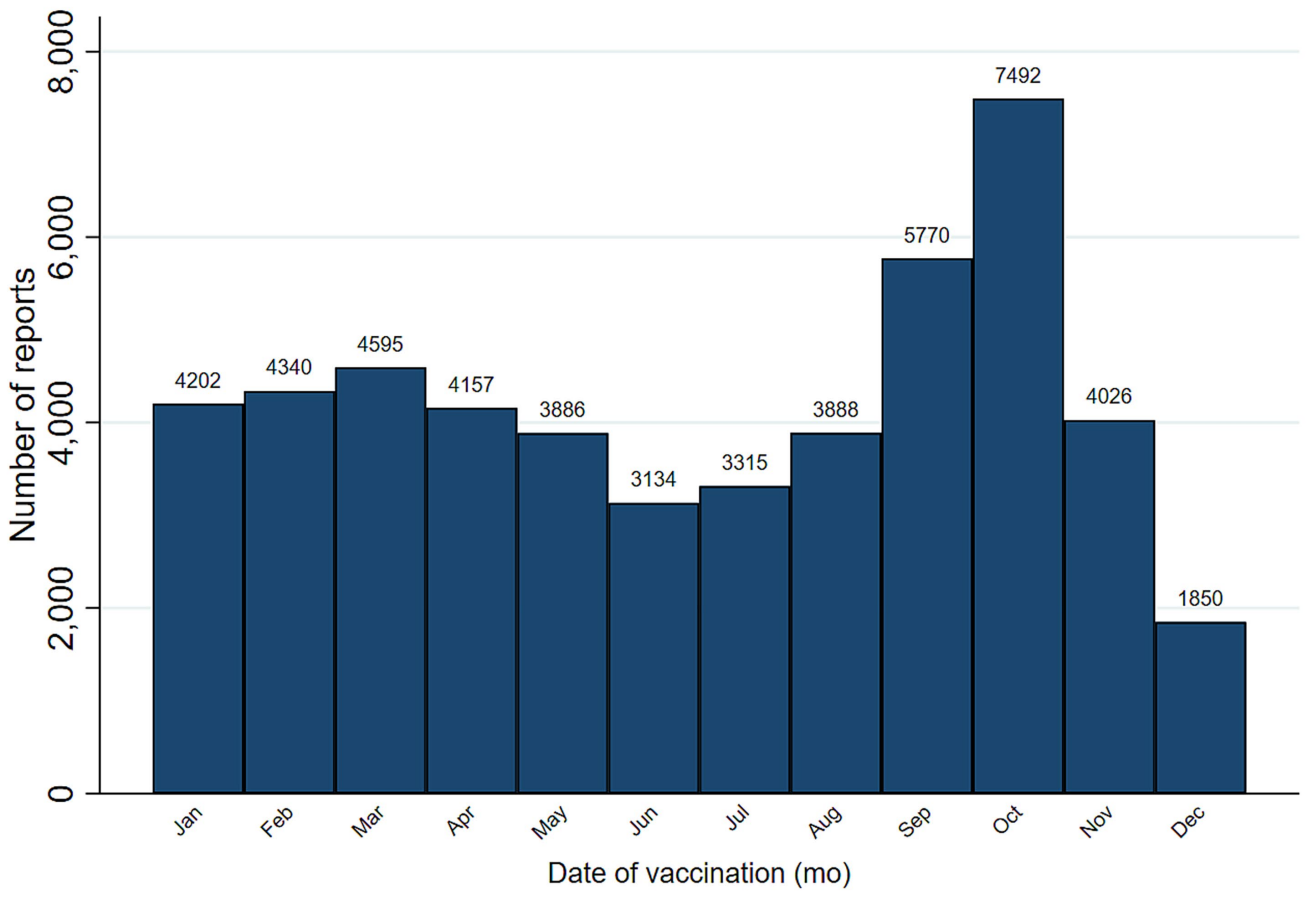
Temporal distribution of VAERS reports by month (*N* = 50,655). The bar chart illustrates the absolute monthly volume of adverse event reports throughout the study period. Bars are formatted in navy with black outlines and zero spacing (gap 0) to highlight the continuous temporal progression. Exact report counts are displayed at the top of each bar for clarity. The horizontal axis displays the month of vaccination, ranging from January through December.

A total of 50,655 AEFI reports were submitted to VAERS in 2025, and their demographic characteristics are presented in Table 1. The median age of the study population was 40 years (IQR: 11–65 years). The majority of reports originated from adults aged 18–64 (30.00%) and female participants (54.47%). While pediatric vaccine reports were evenly distributed by sex, a marked female predominance emerged in adulthood. It persisted among older adults, with women reporting at nearly twice the rate of men in these age groups (Figure 1). Regionally, the South was the most represented region (31.10%). Temporal analysis revealed a stable baseline from January through August, followed by a dramatic surge in autumn, peaking in October with 7,492 reports (a 78% increase over early summer levels) before dropping to a year-low of 1,850 in December (Figure 2).

**Table 1:** Case characteristics and comparison by case severity (*N* = 50,655).

Variable	Overall (*N* = 50, 655)	Non-serious (*n* = 45,802)	Serious (*n* = 4,853)	*p*-value*
Demographic characteristics				
Age (Years), Median (IQR)	40 (11.65)	38 (11.65)	48 (17.68)	<0.001
Age group, *n* (%)				
Pediatric (<18)	14,477 (28.58)	13,400 (29.26)	1,077 (22.19)	<0.001
Adult (18–64)	15,199 (30.00)	13,299 (29.04)	1,900 (39.15)	
Older adult (65+)	10,792 (21.30)	9,504 (20.75)	1,288(26.54)	
Unknown	10,187 (20.11)	9,599 (20.96)	588 (12.12)	
Sex, *n* (%)				
Male	17,437 (34.42)	15,204 (33.20)	3,233(46.01)	<0.001
Female	27,590 (54.47)	25,163 (54.94)	2,427 (50.01)	
Unknown	5,628 (11.11)	5,435 (11.87)	193 (3.98)	
Region, *n* (%)				
Northeast	6,735 (13.30)	6,065 (13.24)	670 (13.81)	<0.001
Midwest	10,014 (19.77)	9,012(19.68)	1,002 (20.65)	
South	15,756 (31.10)	14,357 (31.35)	1,399 (28.83)	
West	10,141 (20.02)	9,196 (20.08)	945 (19.47)	
Territories	187 (0.37)	174 (0.38)	13 (0.27)	
Unknow	7,822 (15.44)	6,998 (15.28)	824 (16.98)	
Case and clinical characteristics				
Onset Time (days), Median (IQR)	0 (0, 1)	0 (0, 1)	10 (1, 30)	<0.001
Administration site, *n* (%)				<0.001
Pharmacy	13,546 (26.74)	12,531 (27.36)	1,015 (20.91)	
Private	12,973 (25.61)	11,521 (25.15)	1,452 (29.92)	
Public	3,536 (6.98)	3,342 (7.30)	194 (4.00)	
Military	782 (1.54)	577 (1.26)	205 (4.22)	
Other	3,899 (7.70)	3,274 (7.15)	625 (12.88)	
Unknown	15,919 (31.43)	14,557 (31.78)	1,362 (28.07)	
Lab/Test evidence, *n* (%)				<0.001
None/Unknown	45,390 (89.61)	42,027 (91.76)	3,363 (69.30)	
Labs reported	5,265 (10.39)	3,775 (8.24)	1,490 (30.70)	
Injection site reaction, *n* (%)				<0.001
No	28,125 (55.52)	24,001 (52.40)	4,124 (84.98)	
Yes	22,530 (44.48)	21,801 (47.60)	729 (15.02)	
Systemic reaction, *n* (%)				<0.001
No	40,214 (79.39)	37,288 (81.41)	2,926 (60.29)	
Yes	10,441 (20.61)	8,514 (18.59)	1,927 (39.71)	
Patient history				
Concurrent medications, *n* (%)				<0.001
None/Unknown	39,508 (77.99)	36,104 (78.83)	3,404 (70.14)	
Yes	11,147 (22.01)	9,698 (21.17)	1,449 (29.86)	
Current illness, *n* (%)				<0.001
None/Unknown	45,405 (89.64)	41,384 (90.35)	4,021 (82.86)	
Yes	5,250 (10.36)	4,418 (9.65)	832 (17.14)	
Medical history, *n* (%)				<0.001
None/Unknown	39,847 (78.66)	36,586 (79.88)	3,261 (67.20)	
Yes	10,808 (21.34)	9,216 (20.12)	1,592 (32.80)	
Prior adverse reaction, *n* (%)				
None/Unknown	49,367 (97.46)	44, 666(97.52)	4,701 (96.87)	<0.001
Yes	1,288 (2.54)	1,136 (2.48)	152 (3.13)	
Allergy, *n* (%)				<0.001
None/Unknown	39,454 (77.89)	35,920 (78.42)	3,534 (72.82)	
Yes	11,201 (22.11)	9,882 (21.58)	1,319 (27.18)	
Vaccine characteristics				
Vaccine type, *n* (%)				<0.001
COVID-19	10,680 (21.08)	8,173 (17.84)	2,507 (51.66)	
Childhood and routine	17,220 (33.99)	16,282 (35.55)	938 (19.33)	
Influenza	7,061 (13.94)	6,650 (14.52)	411 (8.47)	
Varicella-Zoster	7,221 (14.26)	6,966 (15.21)	255 (5.25)	
Pneumococcal	4,074 (8.04)	3,755 (8.20)	319 (6.57)	
Other	4,399 (8.68)	3,976 (8.68)	423 (8.72)	
Vaccine manufacturer, *n* (%)				
mRNA (Pfizer/Moderna)	10,050 (19.84)	7,715 (16.84)	2,335 (48.11)	<0.001
GSK	12,406 (24.49)	11,894 (25.97)	512 (10.55)	
Merck	8,135 (16.06)	7,685 (16.78)	450 (9.27)	
Sanofi	7,059 (13.94)	6,716 (14.66)	343 (7.07)	
Pfizer/Wyeth	3,281 (6.48)	2,971 (6.49)	310 (6.39)	
Other manufacturers	5,316 (10.49)	4,951 (10.81)	365 (7.52)	
Unknown	4,408 (8.70)	3,870 (8.45)	538 (11.09)	
Dose number, *n* (%)				< 0.001
Dose 1	19,506 (38.51)	17,714 (38.68)	1,792 (36.93)	
Dose 2	7,261 (14.33)	6,251 (13.65)	1,010 (20.81)	
Booster (3+)	4,900 (9.67)	4,223 (9.22)	677 (13.95)	
Unknown/Unspecified	18,988 (37.48)	17,614 (38.46)	1,374 (28.31)	
Vaccination route, *n* (%)				< 0.001
Intramuscular	28,285 (55.84)	25,875 (56.49)	2,410 (49.66)	
Syringe/Injection	2,418 (4.77)	1,607 (3.51)	811 (16.71)	
Subcutaneous (SC)	1,876 (3.70)	1,747 (3.81)	129 (2.66)	
Oral (PO)	663 (1.31)	558 (1.22)	105 (2.16)	
Other minor routes	2,472 (4.88)	2,311 (5.05)	161 (3.32)	
Unknown (UN)	14,941 (29.50)	13,704 (29.92)	1,237 (25.49)	
Anatomical vaccination site, *n* (%)				< 0.001
Left arm	17,279 (38.55)	15,949 (39.08)	1,330 (33.13)	
Right arm	9,015 (20.11)	8,186 (20.06)	829 (20.65)	
Other/Nasal/Oral	814 (1.82)	692 (1.70)	122 (3.04)	
Unknown/Missing	17,713 (39.52)	15,979 (39.16)	1,734 (43.19)	

*p-value: Calculated via Rank-sum test for continuous age and Chi-square for categorical variables.

### 3.2 Vaccine characteristics and clinical presentation

As shown in Table 1, childhood and routine vaccines accounted for 33.99% (*n* = 17,220), followed by COVID-19 (21.08%), Varicella-Zoster (14.26%), and Influenza (13.94%). The most frequently reported manufacturer was GSK (24.49%, *n* = 12,406), followed by mRNA (Pfizer/Moderna) (19.84%) and Merck (16.06%). The majority of adverse events occurred following the first dose (38.51%) and were primarily administered intramuscularly (55.84%), on the left arm (38.55%), and at pharmacies (26.74%) or private facilities (25.61%). Symptoms typically appeared rapidly, with a median onset time of 0 days (IQR: 0,1), and 10.39% had diagnostic or laboratory results. Injection site reactions were highly prevalent, occurring in 44.48% of cases, while systemic symptoms were reported in 20.61% of cases. Regarding patient history, only a small portion of individuals reported concurrent medications (22.01%), current illness (10.36%), medical history (21.34%), prior AEFI (2.54%), and allergies (22.11%).

### 3.3 Case severity and patient outcomes

Analysis of 50,655 AEFI reports processed in 2025 revealed that 9.58% (*n* = 4,853) were unique reports classified as SAEs based on the presence of one or more serious criteria, while the majority were non-serious (90.42%). Consistent with the non-mutually exclusive nature of these outcomes, Table 2 details the frequency of each specific endpoint. Permanent disability was the most frequent serious outcome (3.59%, *n* = 1,820), followed by life-threatening illnesses (1.76%, *n* = 891), deaths (1.54%, *n* = 780), and congenital anomalies (0.10%, *n* = 49). Healthcare resource utilization was substantial, with 22.03% (*n* = 11,158) of cases requiring clinic visits and 10.63% (*n* = 5,383) necessitating emergency room assessment. Furthermore, 5.65% (*n* = 2,861) of reports involved hospitalization, and 0.07% resulted in prolonged inpatient care. At the time of filing, 29.68% of cases reported full recovery, 25.16% had not yet recovered, and 45.16% remained unknown. Details are shown in Table 2.

**Table 2:** Case severity and stratified patient outcomes of adverse event reports (*N* = 50,655).

Category	Frequency (*n*)	Percentage (%)
Case severity		
Non-Serious	45,802	90.42
Serious (SAE)	4,853	9.58
SAE-defining outcomes		
Death	780	1.54
Life-threatening illness	891	1.76
Hospitalization	2,861	5.65
Permanent disability	1,820	3.59
Congenital anomalies	49	0.1
Prolonged hospitalization	37	0.07
Healthcare utilization		
Emergency room visit	5,383	10.63
Clinic visit	11,158	22.03
Recovery status		
Yes	15,036	29.68
No	12,744	25.16
Unknown	22,875	45.16

Outcome categories are not mutually exclusive; a single report may represent multiple patient outcomes. Total serious cases (*n* = 4,853) represent unique reports meeting one or more serious criteria. SAE: serious adverse event.

### 3.4 Comparison of demographic and clinical characteristics by case severity

A comparison between non-serious cases (*n* = 45,802) and SAE reports (*n* = 4,853) revealed distinct differences in reporting patterns (Table 1). Given the large sample size, all examined variables reached statistical significance (*p* < 0.001); however, the magnitude of these differences varied across clinical and demographic categories. Reports classified as serious were associated with a notably higher median age (48 years; IQR: 17–68) compared to non-serious reports (38 years; IQR: 11–65). While both groups showed a female preponderance, the proportion of male reports was higher among SAEs (46.01%) than non-serious cases (33.20%).

Regarding clinical profiles, a substantial difference was observed in median onset time: it was considerably longer for SAE reports (10 days) than for non-serious reports (0 days). Furthermore, SAE reports were more than three times as likely to include documented clinical laboratory evidence (30.70% vs. 8.24%), reflecting the increased clinical intensity associated with serious reporting. SAE reports were characterized by a higher prevalence of systemic reactions (39.71% vs. 18.59%), whereas non-serious reports were predominantly characterized by local injection-site reactions (47.60% vs. 15.02%). SAE reports also consistently reflected more complex medical backgrounds. Specifically, the proportion of reports involving concurrent medications (29.86%), current illnesses (17.14%), and relevant medical histories (32.80%) was markedly higher in the serious group than in the non-serious group (Table 1).

Reporting patterns also differed significantly by vaccine type. More than half of the SAE reports involved COVID-19 vaccines (51.66%), compared to only 17.84% of non-serious reports. Similarly, reports involving mRNA manufacturers were nearly three times more frequent in the SAE group (48.11% vs. 16.84%). In terms of the administration sequence, SAE reporting was more common following Dose 2 (20.81%) and Booster doses (13.95%) than following non-serious reports. These findings highlight specific subgroups and vaccine types associated with a higher likelihood of reporting serious adverse events in the VAERS passive surveillance system.

### 3.5 Factors associated with SAEs

A multivariable logistic regression analysis was conducted to identify factors independently associated with SAEs. The results revealed several significant demographic, clinical, and administrative correlates (Table 3). Both adults (aOR: 1.33; 95% CI: 1.16–1.54; *p* < 0.001) and the older adult (aOR: 1.73; 95% CI: 1.49–2.02; *p* < 0.001) had significantly higher odds of SAEs than pediatric patients. Conversely, females were significantly less likely to experience SAEs than males (aOR: 0.72; 95% CI: 0.66–0.79; *p* < 0.001). The facility administrating the vaccination played a critical role, with military (aOR: 1.76; 95% CI: 1.34–2.32; *p* < 0.001) and private sites (aOR: 1.39; 95% CI: 1.23–1.57; *p* < 0.001) showing increased odds of SAE reporting relative to pharmacy settings, while public facilities showed lower odds of SAE reporting. Additionally, each day of increase in onset time was associated with a slight increase in SAE odds (aOR: 1.06; 95% CI: 1.06–1.07; *p* < 0.001).

**Table 3:** Multivariable logistic regression of factors associated with serious adverse events.

Independent variable	aOR	SE	*z*	*P*	95% CI
Patient demographics					
Age group (Ref: Pediatric<18)					
Adult (18–64)	1.33	0.1	3.98	<0.001	[1.16, 1.54]
Older adult (≥65)	1.73	0.14	7.02	<0.001	[1.49, 2.02]
Gender (Ref: Male)					
Female	0.72	0.03	−7.2	<0.001	[0.66, 0.79]
Unknown	0.18	0.05	−6.71	<0.001	[0.11, 0.30]
Region (Ref: Northeast)					
Unknown	1.22	0.12	2.12	0.03	[1.02, 1.48]
Case and clinical characteristics					
Onset time (Days)	1.06	0.01	32.46	<0.001	[1.06, 1.07]
Administration site (Ref: Pharmacy)					
Private	1.39	0.09	5.21	<0.001	[1.23, 1.57]
Public	0.73	0.08	−2.82	<0.001	[0.58, 0.91]
Military	1.76	0.25	4.04	<0.001	[1.34, 2.32]
Other (Combined)	1.69	0.12	7.11	<0.001	[1.46, 1.95]
Clinical labs reported (Ref: No)	2.48	0.13	17.24	<0.001	[2.24, 2.75]
Site reaction reported (Ref: No)	0.35	0.02	−19.52	<0.001	[0.31, 0.39]
Systemic symptoms (Ref: No)	1.72	0.08	11.46	<0.001	[1.57, 1.89]
Patient history					
Current illness reported (Ref: No)	1.51	0.1	6.58	<0.001	[1.34, 1.71]
Medical history reported (Ref: No)	1.24	0.07	4.04	<0.001	[1.12, 1.38]
Prior reaction reported (Ref: No)	0.77	0.09	−2.18	0.03	[0.61, 0.97]
Allergies reported (Ref: No)	1.14	0.06	2.51	0.01	[1.03, 1.27]
Vaccine factors					
Vaccine type (Ref: COVID-19)					
Non-COVID routine (Range)^&^	0.30–0.48	0.49–0.83	7.41–4.38	<0.001	[0.21–0.65]
Manufacturer [Ref: mRNA (Pfizer/Moderna)]					
Pfizer/Wyeth	1.65	0.34	2.44	0.01	[1.10, 2.46]
Unknown	1.48	0.22	2.61	<0.01	[1.10, 1.98]
Vaccine dose (Ref: Dose 1)					
Dose 2	1.33	0.09	4.27	<0.01	[1.17, 1.51]
Unknown	0.89	0.05	−2.12	0.03	[0.80, 0.99]
Route (Ref: Intramuscular)					
Syringe/Injection	2.07	0.15	9.91	<0.01	[1.79, 2.39]
Subcutaneous	1.51	0.21	2.96	<0.01	[1.15, 1.98]
Oral	2.85	0.93	3.2	<0.01	[1.50, 5.41]
Unknown	0.79	0.06	−3.19	<0.01	[0.69, 0.91]
Site (Ref: Left arm)					
Right arm	1.24	0.07	3.84	<0.01	[1.11, 1.39]
Other/Nasal/Oral	1.92	0.58	2.17	0.03	[1.07, 3.45]
Unknown	2.21	0.15	11.37	<0.01	[1.93, 2.54]

^&^Non-COVID Routine Vaccines include the following: Influenza: 0.48 (95% CI: 0.35–0.65); Varicella-Zoster: 0.30 (95% CI: 0.21–0.42); Pneumococcal: 0.41 (95% CI: 0.28–0.61); childhood and routine: 0.30 (95% CI: 0.22–0.41); Other Routine: 0.39 (95% CI: 0.29–0.52).

The following variables were included in the multivariable model but were not shown in the table as they did not reach statistical significance (*p* ≥ 0.05): regions (Midwest, South, and West); Manufacturers (Merck, Sanofi, mRNA [Pfizer/Moderna], and others), vaccine dose [booster (3+)], vaccination routes (other minor routes), and vaccination sites (other/nasal/oral) Model Fit: The final model demonstrated strong predictive power (Likelihood Ratio chi^2^ = 6776.94; *P* < 0.001; Pseudo R^2^ = 0.31). SE, standard error; aOR, adjusted odds ratio.

Clinical indicators were strongly associated with case severity. The reporting of clinical labs (aOR: 2.48; 95% CI: 2.24–2.75; *p* < 0.001) and systemic symptoms (aOR: 1.72; 95% CI: 1.57–1.89; *p* < 0.001) were associated with increased risk of SAE reporting. In contrast, the presence of a site reaction was protective against an SAE classification (aOR: 0.35; 95% CI: 0.31–0.39; *p* < 0.001). Regarding patient history, the presence of a current illness (aOR: 1.51; 95% CI: 1.34–1.71; *p* < 0.001), a reported medical history (aOR: 1.24; 95% CI: 1.12–1.38; *p* < 0.001), and known allergies (aOR: 1.14; 95% CI: 1.03–1.27; *p* = 0.01) were all associated with increased odds of SAEs. Interestingly, a history of prior adverse reactions was associated with lower odds of SAEs (aOR: 0.77; 95% CI: 0.61–0.97; *p* = 0.03).

Vaccine-specific variables showed significant variation. Compared with COVID-19 vaccines, non-COVID routine vaccines were associated with substantially lower odds of SAEs (aOR range: 0.30–0.48; *p* < 0.001), although Pfizer/Wyeth products were associated with an increased risk (aOR: 1.65; 95% CI: 1.10–2.46; *p* = 0.01). The second dose of a vaccine series was also associated with higher odds of an SAE compared to the first dose (aOR: 1.33; 95% CI: 1.17–1.51; *p* < 0.001). Regarding the route of administration, oral (aOR: 2.85; 95% CI: 1.50–5.41; *p* < 0.01), syringe/injection (aOR: 2.07; 95% CI: 1.79–2.39; *p* < 0.001), and subcutaneous routes (aOR: 1.51; 95% CI: 1.15–1.98; *p* < 0.001) were all associated with higher SAE odds than intramuscular injection. Finally, relative to the left arm, vaccination in the right arm (aOR: 1.24; 95% CI: 1.11–1.39; *p* < 0.01) and other/nasal/oral sites (aOR: 1.92; 95% CI: 1.07–3.45; *p* = 0.03) were associated with significantly increased odds of SAEs.

## 4 Discussion

### 4.1 Summary of the findings

This study represents the first comprehensive evaluation of all AEFIs using the most recent national VAERS dataset, providing a critical update to the vaccine safety landscape. Our findings underscore the robust safety profile of the current immunization schedule while identifying specific patterns associated with SAEs. By concurrently analyzing demographic variables, clinical manifestations, pre-existing health status, and administrative and vaccine-specific parameters, this research elucidates the complex, multifaceted drivers of SAEs. Although most AEFIs were non-serious, this study reports a 9.58% SAE rate, indicating a substantial clinical burden. Key factors associated with SAE reporting include advanced age, male sex, and the presence of pre-existing medical conditions or current illness. Vaccine-specific findings indicate that COVID-19 vaccines, second doses, and non-intramuscular administration routes are associated with a higher risk of SAEs. These results underscore the need for enhanced screening and monitoring of high-risk populations to optimize vaccine safety protocols.

### 4.2 SAE reporting rate

Our analysis indicates that although the vast majority of AEFI reports are non-serious (90.42%), the SAE rate of 9.58% warrants significant clinical and public health consideration. This SAE proportion is slightly higher than that reported in other areas, such as China (0.28/100,000) (17), Ghana (0.4%) (18), and the WHO Western Pacific Region (4.1%) (19). However, it should be interpreted as a measure of surveillance sensitivity rather than an indication of elevated vaccine-related harm. Passive reporting systems are effective at catching serious health issues because people are more likely to report them, whereas minor side effects, such as a sore arm or a mild fever, often go unreported (20, 21). This “reporting pyramid” phenomenon concentrates the data pool on high-priority clinical events. Furthermore, the observed 9.58% SAE reporting rate aligns with large-scale analyses from modern passive surveillance systems such as VAERS, which have consistently categorized approximately 6.6 to 9.2% of all adverse event reports as serious (21–23). Thus, our findings align with high-functioning global reporting infrastructures optimized for detecting critical safety signals.

### 4.3 Clinical severity and healthcare utilization

A granular analysis of SAE outcomes in this cohort reveals a substantial clinical and resource burden. Among serious outcomes, permanent disability was the most frequent (3.59%), followed by life-threatening illnesses (1.76%) and deaths (1.54%). While these figures highlight the gravity of the reported events, the relatively high frequency of disability reports warrants longitudinal monitoring to distinguish between transient functional impairment and long-term sequelae (23). This clinical gravity is mirrored by substantial healthcare resource utilization: nearly 22.03% of cases required clinic visits, 10.63% necessitated emergency department evaluation, and 5.65% involved hospitalization. The recovery status at the time of filing—where only 29.68% had achieved full recovery—highlights a critical temporal gap inherent in passive surveillance. The high proportions of “unknown” (45.16%) and “not yet recovered” (25.16%) statuses likely reflect the “snapshot” nature of initial filings, in which reports are often submitted during the acute phase of an event before final clinical resolution is reached (24). This underscores the necessity of integrated follow-up mechanisms to accurately distinguish between ongoing morbidity and delayed recovery in national safety databases.

### 4.4 Factors associated with SAE reporting

Our multivariable analysis identifies a complex interplay of host factors, clinical presentations, and administrative settings that drive SAE reporting. The significantly higher odds of SAEs among adults (aOR: 1.33) and older people (aOR: 1.73) compared to pediatric populations likely reflect age-related physiological vulnerabilities and a higher prevalence of baseline comorbidities (25). Conversely, the finding that females (aOR: 0.72) were less likely to experience SAEs is noteworthy, as women typically report higher rates of overall AEFIs (26). This suggests that while females may experience more frequent reactogenicity, the most severe clinical outcomes in this dataset were disproportionately concentrated among males (25).

Our study showed that each additional day of onset time is associated with a 6% increase in the odds of SAE reporting (aOR: 1.06). It suggests a significant association between symptom latency and the clinical severity of the reported case. This is consistent with the clinical profile of many high-interest adverse events, such as myocarditis or vaccine-induced immune thrombotic thrombocytopenia (VITT), which typically present several days to weeks after the trigger (27). This finding suggests that clinicians should advise patients to remain vigilant beyond the immediate 24–48-h post-vaccination window. Follow-up resources should specifically target the 5–14-day period, when complex, systemic signals—such as myocarditis or delayed inflammatory responses—are more likely to emerge.

The facility type also played a significant role in SAE reporting, with military (aOR: 1.76) and private sites (aOR: 1.39) showing higher odds of reporting than pharmacies. This variation may stem from differences in institutional reporting mandates or the types of populations served by these facilities (28, 29). For instance, military facilities have a robust ‘reporting culture’ and the Department of Defense’s unique readiness requirements, which mandate strict documentation of all medical encounters (28). The disparity in reporting between various facilities suggests a need for standardized reporting tools across all settings. Pharmacists, as frontline immunizers, should be provided with more integrated, automated reporting systems to ensure that their high-volume data is captured with the same granularity as clinical or military environments.

Clinical indicators served as robust proxies for case severity. The presence of clinical labs reported is one of the strongest factors associated with SAE classification, with an odds ratio of 2.48. This reflects the objective nature of SAE, which signifies a more complex clinical encounter that often requires diagnostic confirmation or hospitalization (30). Interestingly, systemic reactions were associated with increased odds of SAE reporting, while localized site reactions (aOR: 0.35) were associated with decreased odds. This reflects the fundamental clinical and regulatory distinctions between these two types of adverse occurrences. Localized reactions (e.g., pain, redness, or swelling at the injection site) are considered “expected” and “non-serious” in most vaccine and drug trials, indicating a healthy, contained immune response (31). In contrast, systemic reactions, such as anaphylaxis, high fever, or organ dysfunction, are inherently more likely to require the medical interventions that trigger mandatory SAE reporting (5).

Regarding patient history, the presence of current illness (aOR: 1.51), medical history (aOR: 1.24), and known allergies (aOR: 1.14) as factors associated with SAE reporting aligns with the “vulnerable host” hypothesis (32). This framework posits that individuals with pre-existing immune dysregulation, chronic disease burden, or heightened allergic sensitivity have a systematically lower physiological threshold for experiencing severe adverse reactions to pharmaceutical or immunological interventions (33). The gradient of odds ratios—with current illness showing the strongest association—further suggests a dose–response relationship between the intensity of pre-existing immune activation and SAE risk. Interestingly, a history of prior adverse reactions (aOR: 0.77) was associated with decreased odds of SAEs. This suggests that individuals with prior reactions may benefit from closer clinical monitoring or “pre-screening” that prevents the escalation of subsequent events (34).

Our analysis identified several vaccine-related factors strongly associated with the likelihood that a report would be classified as serious. Notably, routine vaccines were associated with 52–70% lower odds of being reported as an SAE compared with COVID-19 vaccines (aOR range: 0.30–0.48; *p* < 0.001). This disparity likely reflects a ‘reporting stimulus’ or ‘heightened reporting environment’ rather than a direct measure of increased biological risk. During the study period, COVID-19 vaccines were administered under intense public and regulatory scrutiny. Factors such as mandatory reporting requirements for healthcare providers under Emergency Use Authorizations (EUAs), high-profile media coverage, and increased public awareness likely lowered the threshold for submitting serious reports for COVID-19 vaccines compared to long-established routine programs (16).

Specifically, the higher odds of SAE classification associated with Pfizer/Wyeth products (aOR: 1.65) must be interpreted within this context. While large-scale meta-analyses have quantified specific systemic signals, such as myocarditis or lymphadenopathy for mRNA platforms (35), the increased odds in our model likely capture a combination of these known reactogenicity profiles and a disproportionately high reporting sensitivity. Traditional inactivated or subunit routine vaccines, which are characterized by well-understood and stable safety profiles, exhibited substantially lower odds of serious classification. This underscores the influence of surveillance intensity and reporting behavior on the outcomes captured within passive monitoring systems like VAERS.

Our analysis reveals that administration characteristics are significant determinants of report severity. The increased odds of an SAE following the second dose (aOR: 1.33) align with the anamnestic immune response, where the “boost” phase triggers more vigorous systemic reactogenicity compared to the initial “prime” dose (36). This finding provides a clear benchmark for patient counseling. Healthcare providers can use this data to set realistic expectations regarding reactogenicity in a prime-boost series, potentially improving series completion rates by preparing patients for the likelihood of more robust systemic symptoms.

Furthermore, the finding that non-intramuscular routes—particularly oral (aOR: 2.85) and subcutaneous (aOR: 1.51) administration—carry higher odds of SAEs reinforces the clinical importance of injection depth. Antigens delivered outside the highly vascularized muscle tissue can lead to prolonged inflammatory responses and higher reporting of severe symptoms (37). These results underscore the need for rigorous staff training in administration techniques. Ensuring vaccines intended for IM use are not accidentally delivered subcutaneously can reduce localized “pooling” and subsequent serious reports.

Our model also identified an association between the injection site and the likelihood that a report would be classified as serious, with right-arm vaccination associated with higher odds (aOR: 1.24) compared with left-arm vaccination. One potential, albeit hypothetical, explanation for this finding relates to functional interference. Because the majority of the population is right-hand dominant, adverse reactions in the dominant arm may have a greater perceived impact on activities of daily living than similar reactions in the non-dominant arm (38). Under the regulatory definitions used by VAERS, significant functional impairment can contribute to a ‘serious’ classification. Individuals may be more likely to report an event as serious when it interferes with their ability to work or perform daily tasks. This tendency is more likely when the dominant limb is affected. However, this interpretation remains speculative, as hand dominance is not captured in VAERS. This finding should therefore be viewed as a reporting signal that warrants further investigation into how physical location and functional impact influence the threshold for spontaneous adverse event reporting.

### 4.5 Limitations

The findings of this study should be interpreted in light of several significant limitations inherent to passive surveillance and the dataset’s structure.

First, the nature of spontaneous reporting leads to inherent reporting biases. As a passive system, VAERS is subject to the “reporting iceberg” phenomenon, where mild or common events are likely under-reported, while serious events are subject to “stimulated reporting.” This was particularly evident during the COVID-19 pandemic, where emergency use authorizations and intense public scrutiny lowered the threshold for reporting serious concerns.

Second, and most critically, the associations identified in this study do not represent “risk factors” for vaccine-induced injury. Because VAERS lacks a known denominator (the total number of doses administered), it is mathematically impossible to calculate true clinical risk or incidence rates. Instead, our results represent factors significantly associated with the reporting of serious adverse events. While these signals are vital for identifying vulnerable reporting subgroups and generating hypotheses, they should not be interpreted as measures of absolute clinical risk or proof of causality. Future studies should bridge this gap by utilizing active surveillance (e.g., Electronic Health Record-linked databases) to provide the necessary denominator for true incidence estimation.

Third, data sparsity and incomplete records introduce potential selection bias. A substantial proportion of the dataset contained missing values for age, sex, region, dose number, and vaccination site. The significant associations found for several “Unknown” categories suggest that cases with incomplete records may systematically differ from those with complete data. This highlights the need for regulatory bodies to implement standardized, mandatory fields to reduce the data gaps that currently hinder granular safety signaling.

Finally, the generalizability of our findings is constrained by institutional and geographical contexts. Variations in healthcare access and regional mandates mean these associations may differ in global settings with disparate surveillance infrastructures or cultural attitudes toward safety reporting. Future research should employ cross-national comparative analyses to help decouple biological reactogenicity from regional variations in healthcare access or institutional reporting mandates.

## 5 Conclusion

This study provides a comprehensive analysis of factors associated with SAE reporting, revealing that a complex intersection of demographic characteristics, clinical presentations, and vaccine-specific factors influences the likelihood that an adverse event will be reported as serious. Our data showed that 9.58% of AEFIs reported to VAERS in 2025 were classified as SAEs. While this represents a substantial number of reports, it should be interpreted as a surveillance signal rather than a measure of true clinical incidence. The findings indicate that SAE reporting odds are disproportionately higher among cases involving advanced age, male sex, and specific clinical markers such as systemic reactions and prolonged onset latency. Furthermore, vaccine-related variables—including mRNA platforms, second doses, and non-intramuscular routes of administration—emerged as key factors associated with increased reporting sensitivity.

These results underscore the importance of proactive monitoring of identified high-reporting subgroups to refine global vaccine safety surveillance. To improve the accuracy of these signals and mitigate the reporting biases inherent in passive systems, existing frameworks should be integrated with active, EHR-based monitoring. Ultimately, our findings provide a data-driven foundation for identifying reporting trends, helping to inform patient counseling and strengthen the overall resilience of national immunization monitoring programs.

## Data Availability

The datasets presented in this study can be found in online repositories. The names of the repository/repositories and accession number(s) can be found in the article/supplementary material.
